# The local co-evolution of firms and governments in the Information Age

**DOI:** 10.1057/s41267-020-00373-3

**Published:** 2020-10-18

**Authors:** Sarianna Lundan, John Cantwell

**Affiliations:** 1 Faculty of Business Studies and Economics, University of Bremen, 28359 Bremen, Germany; 2 Department of Management Studies, School of Business, Aalto University, Espoo, Finland; 3 Rutgers Business School—Newark, Rutgers University, 1 Washington Park, Newark, NJ 07102 USA

**Keywords:** theory of FDI and the MNE, knowledge transfer and innovation, economic geography, business–government interaction, multi-party cooperation

## Abstract

The governance structures of the value-creating activities of MNEs have evolved towards more networked forms that are geographically highly concentrated and involve partnering with diverse actors. The experimentation that takes place within these corporate networks has a parallel on the government side, where subnational governments, and particularly cities as hubs of economic activity, have increased their profile and level of cooperative activity. We argue that engagement in these partnerships is an essential way in which firms and governments co-evolve and create the basis for sustainable economic growth in the Information Age. While the origins of this collaborative form of governance reside in the increasing knowledge intensity of value creation, its implications go far beyond MNE value creation and capture, extending to issues of global governance such as climate change and sustainable development goals. We examine the implications of this process of co-evolution both in terms of the costs of developing the requisite corporate capabilities as well as the legitimacy of these efforts as part of a deliberative democracy.

## Résumé

Les structures de gouvernance des activités de création de valeur des EMN ont évolué vers des formes plus réticulaires qui sont géographiquement très concentrées et qui impliquent des partenariats avec divers acteurs. L’expérimentation qui a lieu au sein de ces réseaux d’entreprises a un parallèle du côté gouvernemental, où les gouvernements infranationaux, et en particulier les villes en tant que centres d’activité économique, ont accru leur profil et leur niveau d’activité coopérative. Nous soutenons que l’engagement dans ces partenariats est un moyen essentiel pour les entreprises et les gouvernements de co-évoluer et de créer les bases d’une croissance économique durable à l’ère de l’information. Bien que les origines de cette forme de gouvernance collaborative résident dans l’intensité croissante des connaissances en matière de création de valeur, ses implications vont bien au-delà de la création et de la récupération de valeur par les EMN, s’étendant aux questions de gouvernance mondiale telles que le changement climatique et les objectifs de développement durable. Nous examinons les implications de ce processus de co-évolution à la fois en termes de coûts de développement des capacités d’entreprise requises, ainsi que de légitimité de ces efforts dans le cadre d’une démocratie délibérative.

## Resumen

Las estructuras de gobernanza en las actividades de creación de valor de las empresas multinacionales han evolucionado hacia formas más interconectadas que están altamente concentradas geográficamente y que implican asociarse con diversos actores. La experimentación que se lleva a cabo dentro de estas redes corporativas tiene un paralelo con el lado gubernamental en donde los gobiernos sub-nacionales, y en particular las ciudades como centros de actividad económica, han aumentado su perfil y nivel de actividad cooperativa. Argumentamos que la participación en estas asociaciones es una manera esencial en que las empresas y los gobiernos co-evolucionan y crean las bases para el crecimiento económico sostenible en la Era de la Información. Mientras que los orígenes de esta forma colaborativa de gobernanza residen en la creciente intensidad de conocimiento de la creación de valor, sus implicaciones van más allá de la creación y captura de valor de las empresas multinacionales, extendiéndose a los asuntos de gobernanza global como son el cambio climático y los objetivos de desarrollo sostenible. Examinamos las implicaciones de este proceso de co-evolución tanto en términos de los costos de desarrollar las capacidades corporativas necesarias, como de la legitimidad de estos esfuerzos como parte de una democracia deliberativa.

## Resumo

As estruturas de governança das atividades de criação de valor de MNEs evoluíram para formas mais interligadas que são geograficamente altamente concentradas e que envolvem parcerias com diversos atores. A experimentação que ocorre dentro dessas redes empresariais tem um paralelo no lado governamental, onde governos subnacionais, e particularmente cidades como pólos de atividade econômica, têm aumentado seu perfil e nível de atividade cooperativa. Argumentamos que o envolvimento nessas parcerias é uma forma essencial pela qual empresas e governos coevoluem e criam a base para crescimento econômico sustentável na Era da Informação. Embora as origens dessa forma colaborativa de governança residam na crescente intensidade de conhecimento da criação de valor, suas implicações vão muito além da criação e captura de valor da MNE, estendendo-se a questões de governança global, como mudanças climáticas e metas de desenvolvimento sustentável. Examinamos as implicações desse processo de coevolução, tanto em termos dos custos de desenvolvimento das capacidades corporativas necessárias, como da legitimidade desses esforços como parte de uma democracia deliberativa.

## 摘要

跨国公司创造价值活动的治理结构已发展为更加网络化的在地理上高度集中并涉及与不同参与者合作的形式。在这些公司网络内进行的试验在政府方面是平行的, 这对地方政府, 特别是作为经济活动中心的城市, 提高了合作活动的形象和水平。我们认为, 参与这些伙伴关系是公司和政府共同发展并为信息时代可持续经济增长奠定基础的必不可少的途径。尽管这种协作式治理形式的起源在于价值创造的知识强度的提高, 然而它的意义远超出了跨国公司的价值创造和获取, 而延伸到全球治理问题, 例如气候变化和可持续发展目标。我们从开发所必要的公司能力的成本以及作为协商民主的一部分的这些努力的合法性这两个方面研究了这个共同发展过程的意义。

## INTRODUCTION

The past half-century has witnessed a massive transformation of the global economy, as developed economies have shifted from manufacturing to service-driven economies where innovation and knowledge intangibles are essential for value creation, and some of the most dynamic parts of the economy are related to digital services (Branstetter, Glennon, & Jensen, 2018). Research in the field of international business (IB) has also been transformed during that time, with much attention being paid to the changes in the governance structures of the multinational enterprise (MNE) that have transformed it from a centralized and home-country dominated enterprise to a multicenter network enterprise where the corporate headquarters coordinates both the internal and external networks that comprise the value-creation system.

While intrafirm trade (internalized transactions across borders within the MNE) continues to play an important role in world trade, both global merchandise trade and trade in commercial services actually shrunk in 2015–2016, although both recovered past their earlier levels prior to the onset of the COVID-19 pandemic.^1^ At the same time, while MNEs have taken advantage of the opportunities offered by the ‘fine-slicing’ of the value chain, the past decade has been marked by a rise in both left- and right-wing populist politics and politicians, at least some of whom are hostile to an open global economy and the flows of capital and people this entails (Devinney & Hartwell, 2020; Rodrik, 2018).

The goal of this paper is to ask what the shift into the Information Age implies for our understanding of the impact of MNE activity on the home and host countries. We propose that the shift in focus from tangible capital investments (which of course do include intangibles) to investment in new types of knowledge-intensive intangibles has profound implications for governments, as well as for the role of MNEs as corporate citizens. We build our argument by reference to recent research that looks at different forms of public–private partnerships. For example, Lorenzen, Mudambi & Schotter (2020) propose local entrepreneurial development as a strategy to mitigate the costs of extreme concentration at the core locations and the impoverishment of peripheral locations that are associated with cross-border MNE activity. While Lorenzen et al. (2020) look at this strategy of ‘local spawning’ mainly from the point of view of diversifying local entrepreneurial efforts, and by so doing developing a socially sustainable business model, we extend this discussion to examine how local spawning fits into a broader conception of corporate citizenship that we argue is necessitated by the shifts in the global economy.

Our argument proceeds as follows. We begin by taking a look back at one of the earliest empirical studies in IB, which was a study by Dunning on the impact of American manufacturing investment in Britain (Dunning, 1998 [1958]). This pioneering study highlighted the importance of the productivity advantages that were transferred as a result of MNE investment and how this contributed to the upgrading of British industry. We then turn to examine how the shift to the Information Age has changed our understanding of the kinds of advantages that are relevant from the host-country perspective. This change is particularly evident in the concentration of knowledge-intensive activities within a relatively small number of locations globally. At the same time, in a distributed model of innovation, knowledge is not simply carried from the home countries to the host countries, but investment in the development of intangibles takes place in multiple locations that are connected within the MNE.

We then argue that the experimentation that takes place within these corporate networks has a parallel on the government side, where subnational governments, and particularly cities as hubs of economic activity, have increased their profile and level of cooperative activity (Acuto & Rayner, 2016; Côté, Estrin, & Shapiro, 2020). This is manifested in city networks that are looking to forge new types of relationships both horizontally and vertically, i.e., both across borders between cities and between cities and other actors, including MNEs.

We develop this argument within the co-evolutionary framework of Cantwell, Dunning & Lundan (2010) where both firms and governments are seen as adjusting to the shifts in the global economy by engaging in experimentation and generating increasing variety of new governance forms. We will argue that engagement in these partnerships is an essential way in which firms and governments create the basis for sustainable economic growth in the Information Age. While the origins of this collaborative form of governance reside in the increasing knowledge intensity of value creation, its implications go far beyond MNE value creation and capture, extending to issues of global governance such as climate change and sustainable developmepnt goals. We conclude by discussing the implications of co-evolution both in terms of the costs of developing the requisite corporate capabilities, as well as the legitimacy of these efforts as part of a deliberative democracy.

## PRODUCTIVITY AND OWNERSHIP ADVANTAGES

While research that is relevant to what we today consider international business was present before Dunning’s 1958 study, it was fragmented. Although a distinction was made between foreign direct investment (FDI) and portfolio investment (which Hymer (1976 [1960]) was about to render as explicitly central to subsequent IB analysis), the controlling aspect of MNEs was not present in most of the early studies (Buckley, 2011). By reference to carefully collected empirical evidence, Dunning’s pioneering study demonstrated that American investors in British manufacturing had notable advantages as compared to the indigenous firms.

In his study, Dunning (1998 [1958]) presented systematic empirical evidence of the sectors in which American MNEs were active, how their size and other characteristics compared with those of the domestic firms, and how their management and organizational systems differed from their British counterparts. Based on this evidence, he was able to draw some fundamental insights that would come to inform his later development of the eclectic or OLI paradigm. Beyond demonstrating the quantitative significance of this investment for the British economy, Dunning asked the crucial question of what it was that allowed the American firms to outcompete British firms in Britain? After all, it would be reasonable to assume that any domestic firm would have an advantage in its home market vis-à-vis a foreign rival that had less information about the state of the current competition, networks of suppliers, methods of distribution, and available financing, factors which later on came to be known as the liability of foreignness (Hymer, 1976 [1960]; Zaheer, 1995).

Although Dunning was not focused on measuring productivity differences as such, the data he collected revealed it was a productivity advantage (broadly understood) that was fundamental to the ability of the American investors to outcompete the British firms. While some American firms were clearly utilizing superior (proprietary) technology, others gained an advantage through effective marketing and organizational systems that were transferred in whole or in part to their operations in Britain. This benefited British consumers, but it also resulted in productivity improvements in many industries in Britain.^2^

From this initial realization, we can draw a line to the subsequent development of the theory of the MNE, and particularly internalization theory (Buckley & Casson, 1976; Dunning, 1980). If the American firms had a productivity advantage over British firms, it would stand to reason that this advantage was first developed in the United States before being transferred to the subsidiaries of American MNEs in Britain. Early scholars of international business focused on technological product and process improvements that resulted in greater efficiency, reflecting in part the focus at the time on manufacturing industries. Explaining the transfer of technological assets and the trade in intermediate goods associated with these knowledge transfers became the focus of theorizing about the essence of the MNE.

However, empirical investigations also demonstrated that MNEs (firms that owned or controlled value-adding activity in more than one country) were disproportionately present in industries that had a higher intensity of research and development (R&D), but also a higher advertising intensity (Morck & Yeung, 1991). Thus, while some of the knowledge intensive assets were technological, and in some industries also patentable, other advantages had more to do with branding and marketing. Additionally, MNEs often benefited from the exploitation of a scale-based advantage and market power, an aspect of the multinational firm emphasized in the early theory of Hymer (1976 [1960]).

An interesting but often-neglected feature of the early discussion on ownership advantages was that they were defined as advantages due to the nationality of ownership of the firm, drawn from the conditions in its domestic home economy, including any natural or created resources as well as competitive structure (Dunning, 1980). These advantages were then utilized by the firm to develop a productivity advantage. While some advantages such as proprietary technology would clearly be firm-specific, in other words not available to other firms, other aspects of ownership advantages were drawn from elements that at least in principle were openly available to other firms originating and embedded in the institutional environment of the home country.^3^ In subsequent iterations of the eclectic paradigm this distinction became less prominent (Dunning, 1998: 265) as MNE networks grew in maturity and complexity.

Economic theory suggests that since there are costs related to the need to overcome the liability of foreignness, internationalizing firms need to possess a productivity advantage to cover these costs (Helpman, Melitz, & Yeaple, 2004). Specifically, it is expected that since FDI presents even greater costs and risks than exporting, firms that self-select to becoming multinational should be more productive than firms that export, and firms that are more multinational should be more productive than those whose global footprint is smaller (Yeaple, 2009). The empirical evidence concerning productivity advantages reviewed by Dunning & Lundan (2008) is remarkably consistent with this ‘pyramid of productivity’, and it was also confirmed for the US by Mataloni (2011).

From this, it follows that multinational affiliates are nearly always likely to be more productive than the average local firm in the host country, but they are not necessarily more productive than comparably sized domestic firms in the same industry, or indeed indigenous multinationals, particularly in a developed country (Bellak, 2004). Nonetheless, the productivity advantage has been one of the principal reasons for countries to try to attract FDI, and to prefer foreign investment over domestic investment in the hopes of technology transfer through linkages or spillovers to local firms (for reviews of this substantial literature, see e.g., Giuliani & Macchi (2014), Javorcik (2015) and Narula & Pineli (2019)). This process of self-selection and the resulting productivity advantage has also been used to explain why MNEs could afford to pay higher wages than comparable local firms (Driffield, 1996; van der Straaten, Pisani, & Kolk, 2019).

## FROM KNOWLEDGE TRANSFER TO KNOWLEDGE INTEGRATION

Central to the role of MNEs as innovative engines in the global economy is the transfer of knowledge-intensive assets from the home country to the host country. For the MNE, this entails the international dissemination of parts of its core knowledge base in order to sustain what may be termed competence exploiting (CE) activities through adaptation to various foreign contexts (Cantwell & Mudambi, 2005). This is complemented by the generation of new knowledge-based assets in host countries with the possibility of transferring them back to the home countries. A key development in recent times has been the increasing role of MNEs as international knowledge integrators, whereby core business knowledge grounded in home-country networks is combined selectively in host countries with local knowledge due to competence creating (CC) activities which build capabilities for new applications that extend the traditional domains of expertise of the firm (Scalera, Perri, & Hannigan, 2018). In this way, firms and locations co-evolve with each other, and this process of knowledge development and transfer generates economic growth (Cano-Kollmann, Cantwell, Hannigan, Mudambi, & Song, 2016; Freeman, 2013).

CE capabilities are distributed and coupled within the MNE through FDI, as they always were. Thus, FDI retains a critical role in the more geographically and organizationally dispersed structures of international knowledge development that support higher levels of productivity or productivity growth today. However, CE efforts which adapt and extend the core knowledge base of the MNE have now been incorporated into a wider system of interconnected knowledge networks. Given the rising complexity and increasingly interdisciplinary character of technological knowledge, innovation networks have become more open and informal in nature (Cantwell & Salmon, 2019). Building relational capabilities in networks has become more critical to sustaining ownership advantages (Dunning, 1995), and MNEs endeavor to move from positions of outsidership towards insidership in the relevant networks (Johanson & Vahlne, 2009). International knowledge networks have also become denser and rely especially on connections between major hubs, which are the more central network nodes that now include some actors in emerging markets as well. From an MNE perspective, such increasing knowledge interchanges have helped to create the necessary environment for the development of CC capabilities concentrated in selected subsidiaries or closely networked partners in selected host sites.

The traditional view of knowledge flows within the MNE is essentially based on CE activities, which emphasize the commonality of knowledge development and the sharing of knowledge in use, supported by communities of practice in the MNE (Kogut & Zander, 1993; Nohria & Ghoshal, 1997). Yet, innovative CC initiatives are also built upon this foundation of the internal exchange of CE knowledge held in common, as well as on the advantages of external networks generally grounded in the host location. CC efforts rely on novel combinations of CE inherited knowledge and newly discovered knowledge derived from local network connections. So even with the emergence and growth of some CC driven network nodes that move into new areas of application, there is a continuing rationale for an integrated MNE reliant on FDI. Within the ownership-based structures of an MNE, the sharing of CE capabilities has become the glue that holds a now wider relational system together. There is a cumulative interaction between that CE base and the development of novel CC capabilities around selected central nodes, which feeds back into enhancement of the CE core by facilitating an expanding range of new domains of activity.

The international knowledge flows long associated with FDI are now thus part of a system of complementary knowledge exchanges through network linkages. In relative terms, given their CE foundation, intra-MNE transfers between the home and host countries tend to be more focused on knowledge exchange within the core technological fields of expertise of the parent company (Cantwell & Zhang, 2011; Frost, 2001; Zhao & Islam, 2017). Conversely, since subsidiary CC activities by definition delve into new areas of capability building and so move into some new technological domain (Blomkvist, Kappen, & Zander, 2010), they are relatively more likely to exchange knowledge across technological fields through inter-organizational networks. Because CC development typically combines the established branches of MNE knowledge with knowledge acquired through a host-country network, it is in CC contexts in which a need most often arises for subsidiary dual embeddedness (Figueiredo, 2011; Marin, 2006). Although the combination of global integration and local responsiveness is known to give rise to tensions, achieving this balance has become vital for MNE CC activities in innovation hubs. When subsidiaries enter into a new domain for the MNE, this is precisely when their need increases for mutual knowledge interchanges with their parent company and home country, since they must often also expand upon core business knowledge in the new domain of application (Cantwell & Piscitello, 2015). If the new area of application appears promising the parent company is more likely to pay attention, so the exchange is more likely to be encouraged from their side as well and to become reciprocal (Monteiro, 2015).

## GEOGRAPHY OF VALUE CREATION

Now that MNEs are more often positioned as international knowledge integrators within network structures, their development of ownership advantages has shifted in two ways. First, their capability building now relies to a greater extent on some key host-country hubs as well as the parent company in the home country, even if generally the home base remains the single most important location for innovation in the MNE (Patel & Pavitt, 1991). Within these selected central network nodes subsidiaries are more geared towards local CC activities, so they might be termed superstar subsidiaries (Blomkvist et al., 2010). Second, the knowledge sourcing that supports MNE innovation increasingly relies on the integration of local and global knowledge, which in turn entails more systematic knowledge-based interactions between the relevant actors in the host country and those in the home country (Alcácer, Cantwell, & Piscitello, 2016). While in relative terms CC subsidiary inventions depend on a higher share of local knowledge sources compared to CE inventions (Cantwell & Mudambi, 2011), CC inventions also depend on a wider variety of knowledge sources in general, and so they are more likely to combine local with international knowledge.

In the past, the ownership advantages of MNEs were mainly developed in the home country, or arose through the hierarchical coordination of international activities by key agents in the corporate headquarters. Once such advantages had been developed, they were potentially transferrable for use elsewhere, sometimes even throughout the MNE. The local CE use of these capabilities in a host-country context may have then led to further creative development in some initially unanticipated new directions, and hence to feedbacks to innovation in the parent company (Cantwell, 1989). However, the new and ever closer relationship between technology creation and transfer between the central nodes of MNE networks has rendered the distinction between knowledge creation and exchange more ambiguous still, since these more open networks depend on continuous mutual reciprocity. This activity may not always reach as far as the international co-development of technology of the kind that is emphasized in the literature on international inventor teams (Branstetter, Li, & Veloso, 2015; Crescenzi, Nathan, & Rodríguez-Pose, 2016; Hannigan, 2016). However, reciprocal knowledge exchange increasingly occurs in internationalized epistemic communities that are especially well represented in the places (usually metropolitan areas) in which parent company and CC subsidiary innovation facilities are most often sited (Bathelt, Cantwell, & Mudambi, 2018). As mentioned already, these headquarters and central CC subsidiary innovation hubs are of necessity also more often engaged in bidirectional knowledge exchange, in a process through which local and global knowledge is combined and integrated.

The interconnected knowledge networks that MNEs now coordinate or in which they are engaged require more decentralized governance structures and greater informality to function effectively. The span of MNE orchestration and control has become far more widespread than the span of ownership or FDI, and connects selected parts of the MNE with selected external actors, including some nonmarket actors such as universities (Bathelt, Malmberg, & Maskell, 2004; Cantwell & Salmon, 2019; D’Este, Guy, & Iammarino, 2013; Dhanaraj & Parkhe, 2012). However, while more open and internationally connected knowledge networks have become vital for sustained innovation and hence for new value creation, they have given rise to ever greater tension with especially financial and rent-seeking interests more focused on value capture, which continues to rely on the ownership of assets, including intellectual property assets. More mercantilist-oriented states have become involved in the growing conflicts between the demands of value creation and value capture, in which intellectual property policies are generally the result of having to maintain a delicate balance among various stakeholders (Zhao, 2020). The balance to be struck between these competing interests parallels the long recognized balance between broad patent scope (which may favor value capture) and narrow patent scope (which may favor licensing and exchange arrangements that facilitate continued innovation and value creation) (Merges & Nelson, 1990).

In order to benefit from innovation and value creation, home countries need to rely on an open and connected knowledge system that ensures knowledge inflows and reciprocal exchange especially with other key nodes or hubs in the relevant industry. Particularly in recent times, outward FDI has raised incomes in the metropolitan areas in the US, with a cumulative effect that has been strongest in medium-to-high income cities, due to the advantages of greater international knowledge connectivity (Buchholz, Bathelt, & Cantwell, 2020). Among global cities, there is a positive association between international knowledge linkages and local knowledge connections within metropolitan areas, as measured by patent citations (Cantwell & Zaman, 2018). In other words, global and local knowledge networks are increasingly intertwined, and prominent in the interface between the two are MNE parent companies, who now commonly play a brokerage role and thereby benefit themselves from the opportunities for new knowledge combinations and further development.

Meanwhile, host locations need the flexibility to respond to local diversification opportunities as they arise. These are based on potential combinations of the knowledge brought through inward FDI and local strengths, through the creation of new areas of technological relatedness. This is essentially what CC subsidiary activities set out to achieve, and so where successful, these imply not just embeddedness in established local networks, but also the building of new kinds of knowledge network relationships. Yet, as MNE subsidiaries sometimes struggle to become insiders in local knowledge networks, there is now a sharper divergence between those struggling units, and other subsidiaries that evolve into local centers of excellence or knowledge hubs. Selected, more CC-oriented subsidiaries acquire much greater autonomy and dual embeddedness, while others remain essentially MNE satellites. Although the MNE and its FDI are a central and necessary part of the distributed innovation transmission structure for all subsidiaries through CE knowledge flows, in peripheral host locations the benefits are more limited, and such network nodes may be excluded from frontier knowledge exchanges (Monteiro, Arvidsson, & Birkinshaw, 2008). While MNEs have become key international knowledge integrators, in this process there are typically in-groups and out-groups in terms of participation within the MNE (Blomkvist, Kappen, & Zander, 2019).

An increasing geographic unevenness of development is a consequence of these trends, and the locations that benefit the most are more internationally dispersed, particularly metropolitan areas. The key centers also vary across industries, and the spatial structures of their knowledge networks differ across industries (Mudambi, Li, Ma, Makino, Qian, & Boschma, 2018). Core locations tend to have more open knowledge networks, with more inter-cultural exchanges across technical, functional, and social boundaries, in which it is potentially easier for MNE subsidiaries to transition to positions of local insidership. The central nodes of the MNE network are those that develop the capabilities to integrate different strands of knowledge, and thereby to connect their CC and CE activities. By contrast, in other locations, and with some MNE suppliers, there may be less encouragement and even deterrents to knowledge transfer and learning (Alcácer & Oxley, 2014). So, the productivity advantages of the MNE remain, but they now derive primarily from innovation supported by capabilities for knowledge integration developed in the home-country center and in a few other well-connected central hubs.

Research in economic geography has demonstrated that foreign investment in manufacturing is highly location specific and benefits from agglomeration economies (Beugelsdijk & Mudambi, 2013; Dunning & Lundan, 2008: 594). As services have become an even more important component of cross-border flows of investment, it has become evident that knowledge-intensive service activities such as financial services tend to cluster at a similar or even higher intensity than manufacturing investment (Rosenthal & Strange, 2020). Since various knowledge-intensive clusters (combining both services and manufacturing) are the dynamic backbone of developed economies in the Information Age, this has raised concerns about congestion in the selected hotspots that is manifested in overheated labor markets, housing price increases and longer commuting times (Becker, Driffield, Lancheros, & Love, 2020; Kerr & Robert‐Nicoud, 2020).

Beginning with the financial crisis and its aftermath, the global economy has witnessed multiple shocks, which have intensified anti-globalization populism on both the political left and right (Devinney & Hartwell, 2020; Rodrik, 2018). In manufacturing industries, skill-biased technological change has resulted in low to medium-skilled jobs being either automated or offshored to emerging markets. The highly educated knowledge workers that participate in the distributed networks of learning coordinated by multinational firms in core locations have benefited, while those in the periphery risk being disconnected (Prashantham & Bhattacharyya, 2020; Turkina & Van Assche, 2018). Most recently, the COVID-19 pandemic has pushed national governments to experiment with both public health and economic recovery programs of unprecedented scale and variety (Kobrin, 2020).

To alleviate these concerns, and to forestall a populist backlash against their activities, Lorenzen et al. (2020) propose that MNEs should adopt a ‘local spawning’ strategy to encourage entrepreneurship in the cluster locations. In essence, they argue that the MNEs’ social license to operate, especially in high clock-speed industries depends on being viewed not as promoting gentrification and increasing inequality, but more as nurturing a new generation of local entrepreneurial ventures. Although not stated exactly in those terms, the implicit view is that with the MNE shifting from a short-term to a long-term view of the sustainability of the core business, local spawning would not only be beneficial to the local community, but it might also spur new knowledge combinations to which the firm then has privileged access.

This is an intriguing proposal, which in its basic contours is similar to the arguments put forward to encourage MNEs to adopt more comprehensive corporate social responsibility policies in the area of environmental management and human rights (see e.g., Kolk (2016) for a review). It is also a proposal that fits with the existing evidence on the effectiveness of place-based policies, whereby investments in infrastructure, training and improved land development appear to offer better results than incentives, and targeting high-tech (but not large) firms yields the best results in terms of local economic development (Bartik, 2020). At the same time, there are important differences which point to the need to develop new governance arrangements involving a broad coalition of national and local governments, MNEs and other stakeholders in a process of co-evolution.

## LOCAL CO-EVOLUTION OF FIRMS AND GOVERNMENTS

The above discussion has illustrated how MNEs have adapted their structures to the demands of the Information Age. This can be understood as an experimental process whereby firms engage in continuous experimentation to find new governance forms that not only change the balance between equity-based and contractual modes, but also diversify the kinds of partners who get involved. Over time, these adjustments have resulted in a geographically concentrated MNE footprint, particularly for knowledge-intensive activities, that nonetheless also spans the global economy through different kinds of network relationships. The multiple embeddedness of MNE subsidiaries in a variety of local contexts has necessitated the development of operational capabilities to coordinate the intra- and inter-firm network relationships (Meyer, Mudambi, & Narula, 2011).

At the same time, local governments have entered into a type of policy vacuum left by national governments that are locked into a multilateral system ill-equipped to deal with the challenges of the Information Age. The inability of the multilateral system to deal with some of the most pressing transnational issues such as climate change has led governments at the subnational level to take action to fill this institutional void by setting local emissions targets and establishing carbon trading markets (Acuto & Rayner, 2016). Cities have also entered into economic diplomacy on their own and by forming issue-driven collaborative networks (Côté et al., 2020).

While partnering with nonmarket actors is not new to MNEs (Teegen, Doh, & Vachani, 2004), the extent and variety of partnering is changing our understanding of the ownership advantages required for value creation and capture. Dunning & Lundan (2008) identified the role of institutions and institutional change as major challenges for the theory of the MNE. This was based on an understanding that the activities of MNEs in terms of their location or the modality of the activity would no longer be simply determined by the costs and benefits related to the market context, but that they were also increasingly being shaped by significant challenges and uncertainties arising from nonmarket actors.

Their point of reference was the increasing interest in the social performance of MNEs that began with the studies on environmental management in the wake of the of the United Nations Rio conference in the early 1990s. This subsequently widened into the broader agenda of corporate social responsibility, and most recently to the human rights obligations of MNEs, as outlined by Kolk (2016). While some of this research has looked at social engagement from the point of view of minimizing threats and uncertainty in the long run, other areas of research have been focused on examining the possibility of new value creation. In the environmental issues sphere, the participation of MNEs in the regulatory process has become widely legitimated, as these are often problems related to resource efficiency and process technology, where the innovative role of (proactive) MNEs is instrumental to generating solutions (Dowell, Hart, & Yeung, 2000). In other areas, such as when MNEs have been exposed in the media concerning poor labor practices in value chains, their strategy has been to self-regulate to stave off regulation that might otherwise be imposed on them by governments (Graham & Woods, 2006). Consequently, MNEs are seen not only as rule takers but in some ways also as rule makers, though depending on the type and location of activity (Lundan & Muchlinski, 2012).

The more MNEs forge partnerships that allow them to act as rule makers by influencing the content of regulation or setting standards, the more these processes can be interpreted as processes of co-evolution, as envisioned by Cantwell et al. (2010). This view emphasizes the fundamental nature of MNEs as the innovative engines in the global economy. Yet, this innovativeness relies on an embeddedness in knowledge networks that reach into many other parts of society, including universities and research institutes, as well as civil society. Relatedly, this innovative engine is not only focused on generating innovations that increase productivity in the conventional production sphere, but it is also an innovative engine that generates new forms of governance and relationships. This dual nature of MNEs as both technological and governance innovators makes them critical players in responding to many of the transnational challenges facing the global economy, such as climate change impact mitigation or more broadly the sustainable development goals (Pisani, Kolk, Ocelík, & Wu, 2019; van Zanten & van Tulder, 2018).

To deal with this new reality within the OLI framework, Dunning & Lundan (2008) introduced Oi advantages, which consisted of capabilities needed to manage in the nonmarket domain, and distinguished them from the Oa and Ot advantages, where Oa are the classical asset-based advantages of technologies and brand names, and the Ot advantages are the advantages of common governance of activities inside the firm. In a hypothetical world without external shocks, whether economic, technological, or natural, and with stable institutions, the asset and coordination advantages of multinationals are sufficient to explain their location and internalization decisions. On the other hand, in a complex scenario, where institutional change is rapid or individual actors have considerable influence on the outcomes, creating institutional advantages increases in importance (Lundan, 2010).

Such advantages can of course not simply be assumed into existence, but need to be developed through different forms of multi-stakeholder partnering, including partnering with governments (at all levels) as well as civil society organizations (Dunning & Lundan, 2010; Lundan & Li, 2019). This process is difficult, since the objectives and capabilities of the various parties are quite different, and the joint objective is to develop governance structures under which value can be created and captured (van Zanten & van Tulder, 2018). In addition to combining different sources of knowledge, the process of gaining legitimacy for the resulting governance forms can be challenging.

The idea that firms need to partner with multiple stakeholders to develop the governance structures under which value can be created and captured is not something that has generally been thought of as the responsibility of firms beyond the governance of their own value chains. In a narrow conception of corporate social responsibility, firms are tasked with becoming more attuned to the changing environmental or social preferences of their customers and employees from a profit perspective, but essentially on their own terms. In this conception, what is considered to be in the remit of responsibility, let alone how the results are to be measured and reported, is largely left for the firms themselves to define and implement, as long as they abide by existing laws and regulations and can satisfy the demands of their stakeholders.

By contrast, the concept of deliberative democracy as discussed by Scherer & Palazzo (2007, 2011) or corporate diplomacy as discussed by Henisz (2016) that draw on sociology and political science entail a wider remit of responsibility and a questioning of the legitimacy of different governance structures. For example, while some public–private partnerships in the infrastructure sector are long-standing and have an established contractual form, the kinds of smart city projects exemplified by Sidewalk Labs in Toronto, which ran into difficulties concerning the need to craft new regulations for the digital economy (Côté et al., 2020), or the projects under the Chinese Belt Road Initiative (Buckley, 2020), involve new forms of governance where legitimacy is difficult to establish without a process of continued deliberation. At the same time, such processes of deliberation are likely to be strongly influenced by local institutional conditions as well as the micro-foundations of strategy (Contractor, Foss, Kundu, & Lahiri, 2019), i.e., the preferences and priorities of the individuals spearheading such projects. The notion that firms (or, e.g., city governments) have active and legitimate agency in such processes could be contested, but ultimately it is the types of partnerships forged and the outcomes achieved that matter, and these should be the focus of attention of international business scholars.

## CONCLUSION

The complex effects that investment by MNEs induces on their home and host countries by creating new industries, increasing the efficiency of existing industries, or accelerating the rate of overall investment are traceable back to an initial productivity advantage. Without this advantage it would not be possible for firms to overcome the costs of adjustment and distance that are required to successfully exploit their knowledge-based assets abroad. Furthermore, both the positive and negative effects resulting from MNE activity derive from the unique combination of ownership-specific advantages and market power each firm possesses. These effects were present, at least in a rudimentary form, already in the analysis presented by Dunning (1998 [1958]).

Further research over the past half century has contributed to a substantial body of literature within IB and economics examining the specific contingencies that might lead to both positive and negative outcomes for the home and host countries. Value chains coordinated by MNEs can lead to innovation, industrial upgrading, higher wages and better working conditions, or they can do the opposite. While the positive outcomes can be facilitated by policy (e.g., linkage programs, training, infrastructure, R&D credits), policy failures can also lead to exploitation or overuse of resources, and MNE investment can both accelerate and amplify such failures. We have argued in this paper that MNEs make a contribution to the global economy by experimenting not only with new ways of configuring the value chain, but by developing new governance forms in multi-stakeholder partnerships.

We presented a three-pronged argument. First, we examined how the governance structures of the value creating activities of MNEs have evolved towards more networked forms that are geographically highly concentrated. Second, we expanded on the proposal put forward by Lorenzen et al. (2020) of ‘local spawning’ of entrepreneurial activity by MNEs as a way to alleviate some of the negative social consequences of the high concentration of innovative activity. We connected this proposal to the broader discussion about corporate social responsibility, and outlined some of the governance challenges this model of engagement implies. Third, we proposed that where multinationals engage in local experimentation both in their value creation activities and in terms of governance forms, and where experimentation is also being pursued in parallel by governments, a co-evolutionary framework offers a general model for understanding the dynamics of firm-government relations in the Information Age.

For MNEs this is an unprecedented challenge, as these partnerships take place in a very specific context and require the development of specific skills of partnering. Intra-country differences can be very large, and consequently cities have emerged as the effective level at which these strategies can be crafted and new governance forms developed. We can point to a few examples of these kinds of developments already appearing in the literature. One example is an urban development project led by the Sidewalk Labs unit of Alphabet in Toronto which ran into difficulties in negotiations about new regulations for data governance, intellectual property and privacy, with the project eventually being withdrawn (Côté et al., 2020). In other cases, knowledge-intensive multinationals have attempted entry but have been spurred by local communities amidst fears of increasing inequality and gentrification, as was the case with Amazon’s second headquarters, which was due to be located in New York but was subsequently moved to Virginia. Another example is the attempt by Guggenheim to build a satellite museum in Helsinki, which was seen as elitist and not serving the public interest (Ritvala, Granqvist, & Piekkari, 2020). In all three cases, there was initial support from local government officials, who believed that the projects would contribute to the city in a positive way. However, in the latter two cases this was coupled with a lack of sufficiently nuanced understanding of the broader sensitivities surrounding the project by its main promoters. This blindness (at least in hindsight) on the part of sophisticated actors is indicative of the complexity of this kind of public–private partnering, and it is also a prevalent characteristic in the examples from the mining industry discussed by Henisz (2016).

In the current political and economic climate, there is a distinct possibility that ownership advantages may again become more closely tied to the home country rather than being purely firm-specific. An essential aspect of this techno-nationalism (Petricevic & Teece, 2019) is the increasing involvement of national governments in the organization of economic activity with the goal of not only facilitating value creation, but ensuring value capture by domestic MNEs. Essentially, each country tries to ensure that it occupies the upward parts of the ‘smile curve’ involving R&D, design and marketing, where value capture is the highest (Mudambi, 2008). There is thus an inherent contradiction between the techno-nationalist paradigm and the reality of knowledge creation within multinational firms, that is increasingly dependent on access to localized knowledge sources abroad (Buckley & Hashai, 2020).

At the same time, it is an open question how far the structure of the current global economy is in fact reversible, at least in the short run. The collapse of the first global economy after World War I was rapid and comprehensive, but Kobrin (2017) provides reasons to believe that in the current global economy, which is much more interdependent and much more diverse in terms of the modalities that are used to govern cross-border flows of value adding activity, it is conceivable that the structures are more robust in the face of massive change. The level and location of activity within value chains can be adjusted without necessarily engaging in equity investment or divestment (Cuervo‐Cazurra, Doz, & Gaur, 2020). In another process of experimentation, the various restrictions on travel and migration due to the COVID-19 pandemic are being at least partially overcome by greater digital connectivity by MNEs. These challenges underline the need for MNEs to develop a broad variety of institutional knowledge and capabilities that allow them to partner effectively and build ecosystems that are both socially and economically sustainable.

## Notes

1. Data taken from https://data.wto.org/.
2. The (average) productivity improvements as a result of FDI are generally due to a combination of the exit of underperforming domestic firms (a composition effect) and any improved productivity of the remaining domestic firms (Caves, 1982).
3. Even if the resources are generally available, some firms may have privileged access to them, or they may engage in actions to block the access of other users to these assets (Verbeke, Coeurderoy, & Matt, 2018). This is a feature that has been examined in the host-country context (Hennart, 2009), but that has not received as much attention in the home-country context.
